# Challenges in the development of digital public health interventions and mapped solutions: Findings from a scoping review

**DOI:** 10.1177/20552076221102255

**Published:** 2022-05-26

**Authors:** Ihoghosa Iyamu, Oralia Gómez-Ramírez, Alice XT Xu, Hsiu-Ju Chang, Sarah Watt, Geoff Mckee, Mark Gilbert

**Affiliations:** 1 School of Population and Public Health, 120479 University of British Columbia, Vancouver, BC, Canada; 2 British Columbia Centre for Disease Control, Vancouver, BC, Canada; 3 469220 CIHR Canadian HIV Trials Network, Vancouver, BC, Canada

**Keywords:** Digital public health, digital health, public health, eHealth, mHealth

## Abstract

**Background:**

“Digital public health” has emerged from an interest in integrating digital technologies into public health. However, significant challenges which limit the scale and extent of this digital integration in various public health domains have been described. We summarized the literature about these challenges and identified strategies to overcome them.

**Methods:**

We adopted Arksey and O’Malley's framework (2005) integrating adaptations by Levac et al. (2010). OVID Medline, Embase, Google Scholar, and 14 government and intergovernmental agency websites were searched using terms related to “digital” and “public health.” We included conceptual and explicit descriptions of digital technologies in public health published in English between 2000 and June 2020. We excluded primary research articles about digital health interventions. Data were extracted using a codebook created using the European Public Health Association's conceptual framework for digital public health.

**Results and analysis:**

Overall, 163 publications were included from 6953 retrieved articles with the majority (64%, n = 105) published between 2015 and June 2020. Nontechnical challenges to digital integration in public health concerned ethics, policy and governance, health equity, resource gaps, and quality of evidence. Technical challenges included fragmented and unsustainable systems, lack of clear standards, unreliability of available data, infrastructure gaps, and workforce capacity gaps. Identified strategies included securing political commitment, intersectoral collaboration, economic investments, standardized ethical, legal, and regulatory frameworks, adaptive research and evaluation, health workforce capacity building, and transparent communication and public engagement.

**Conclusion:**

Developing and implementing digital public health interventions requires efforts that leverage identified strategies to overcome diverse challenges encountered in integrating digital technologies in public health.

## Introduction

The potential for digital technologies to improve the delivery and impact of public health interventions on the health and wellbeing of populations and communities is widely acknowledged.^1–3^ Interest in integrating digital technologies in health services has resulted in digital health as a field of practice, which has in turn been further adapted in public health as the evolving field of “digital public health.”^2,4^ The term “digital public health” became widely used after public health England (PHE) described its Digital-First Strategy in 2017, and it has been used in different ways to refer to the integration of digital technologies to advance or reimagine public health goals and functions, maximizing its impact on communities and populations by reaching more people with more efficiently delivered health services.^2–5^ Digital public health has been considered to be a promising strategy to tackle substantial modern public health challenges, including aging populations, the dual burden of noncommunicable and communicable diseases, and the health impacts of climate change, among others.^2^

However, numerous challenges impede achieving these potential impacts of digital public health,^6^ including policy and ethics-related dilemmas and complexities inherent with integrating specific digital technologies in public health.^7,8^ Many of these challenges apply to digital health generally and are not specific to digital public health.^9^ However, public health action requires wide-ranging public engagement, a population health perspective, and prompt information exchange and cooperation across diverse organizations and public health agencies. These characteristics of public health services necessitate thinking specifically about challenges pertinent to integrating digital technologies within public health. Given that digital public health is in its nascent stages of development, it is critical to characterize these challenges and implement strategies to overcome them.^6,7^ Drawing on insights from published literature, we aimed to (1) describe shared challenges to integrating digital technologies across various public health domains, (2) summarize recommendations to overcome these shared challenges, and (3) identify common solutions that can form the core of a digital public health strategy. We hoped such a high-level description may help policy makers, public health practitioners, decision makers, and researchers to facilitate systems-level changes that enable a more considered development of digital public health strategies and services.

## Methods

### Overview

We conducted a scoping review to define the field of digital public health. Our methods and findings about the definition of the field have been described in detail elsewhere.^5,10^ Here we focus on the challenges and recommended solutions to support the development and implementation of digital public health interventions. We followed Arksey and O’Malley's framework for scoping reviews,^11^ with adaptations suggested by Levac et al..^12^ Our reports adhere to the Preferred Reporting Items for Systematic Reviews and Meta-Analyses (PRISMA) for scoping reviews.^13^

### Data sources

Our search strategy (Appendix 1) was applied to MEDLINE [Ovid] and Embase [Ovid] to identify literature containing keywords related to both “digital” and “public health.” Our search strategy explored the intersection between digital health (and closely related domains, e.g. virtual health, mHealth, e-health, digitalization) and public health domains described by the Canadian Public Health Association (CPHA) (e.g. health promotion, surveillance, and epidemiology).^14^ We reviewed publications that conceptually described digital technology in public health including expert opinions, commentaries, and reviews. We excluded primary research studies like trials and cross-sectional studies, given that our focus was on assessing the discourse around digital technologies in public health and not specifically on assessing any digital interventions in public health.^10^ We included articles published in English between January 2000 and June 2020. Included titles and abstracts were exported to Covidence® for further review and citation management.^15^

We also conducted a grey literature search on Google Scholar using the search terms: “digital” AND “public health.” This simplified search strategy was used due to lack of more precise search functions on Google Scholar. We further applied these terms on the Google search engine to inspect 14 pre-identified government and intergovernmental agency websites.^10^ We reviewed the first 100 returns from Google Scholar and each website searched. Finally, manual reference list searches were conducted on included articles to identify additional publications.

### Screening procedure

**Figure 1.**
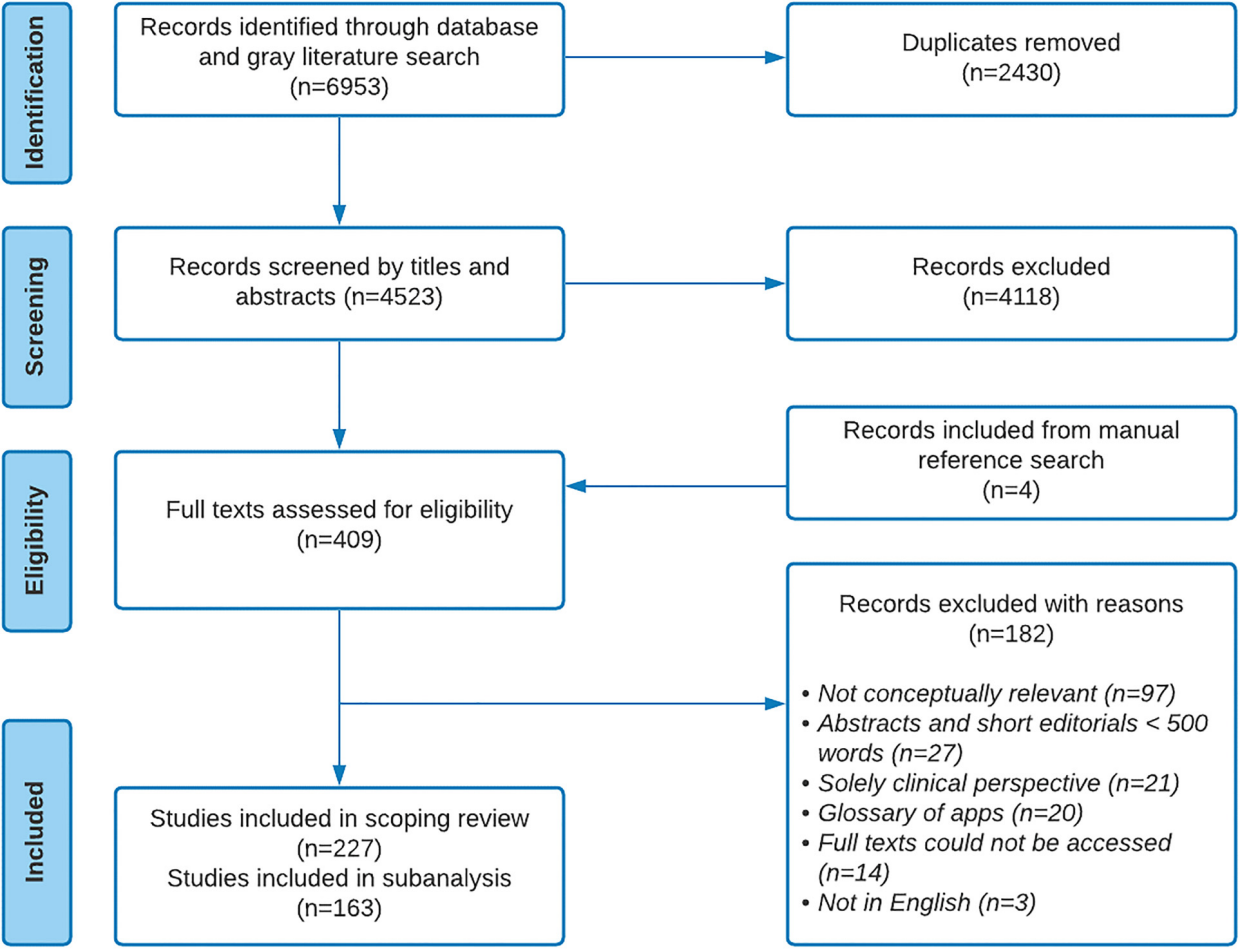
Preferred Reporting Items for Systematic Reviews and Meta-Analyses for Scoping Reviews (PRISMA-ScR) flow diagram of the search.

Pre-established inclusion and exclusion criteria were used during the title and abstract screening.^10^ Articles that broadly conceptualized digital health from a population and public health perspective that were published in English between January 2000 and June 2020 were included (Table 1). We drew on the CPHA's definition of public health as “an organized effort of society to keep persons healthy and prevent injury, illness and premature death, including a combination of programs, services, and policies that protect and promote the health of all.”^14^ Publications that evaluated specific health programs or interventions, focused solely on clinical perspectives, or short summaries of less than 500 words were excluded. Twenty-five percent of the titles and abstracts were independently screened by two reviewers (II and AX) in an iterative approach. Both reviewers met frequently to discuss discrepancies and achieve consensus. Once a general understanding of the procedure was achieved, the remaining titles and abstracts were screened by at least one reviewer. In the full-text screening, all included publications were independently screened by both reviewers using a structured framework (Appendix 2). All discrepancies were discussed among both reviewers until a consensus for inclusion or exclusion was achieved. For this analysis, we identified articles that broadly described challenges to the integration of digital technologies in various public health domains; and/or made recommendations about ways to effectively support the development and implementation of digital technologies in public health (a summary of the selection process is described in Figure 1).

**Table 1.** Inclusion and exclusion criteria.

Parameter	Inclusion criteria	Exclusion criteria
Phenomenon of interest	Publications that broadly conceptualize or analyze digital health from a public health perspective	Publications evaluating or describing specific digital health programs or interventions
Health context	Publications focusing on health issues at the population level including perspectives on preventive, community medicine, or public health (e.g. environmental health, obesity, diabetes, stigma, antibiotic resistance, prevention of sexually transmitted, and blood-borne infections)	Publications solely focused on the application of digital health in clinical contexts
Language	English	Not in English
Publication status	Published or grey literature	No full text, only abstract or short summary <500 words published
Year of publication	January 2000–June 2020	None

### Data extraction and analysis

We imported included publications into QSR NVivo version 12^TM^ and two reviewers (II and AX) independently extracted bibliographic characteristics including article type, publication year, country, and continent of institutional affiliation of the first author. Challenges described in developing digital technologies in public health and solutions recommended were extracted for each publication. Thereafter, we applied a thematic analysis to the text from included papers, following Braun and Clarke's recommendations for thematic analysis.^16^ First data familiarization was done to understand the general challenges and recommendations discussed. Thereafter, we generated the initial codes using inducive techniques that were grounded in the data. We used similar or the same words used in the articles to generate the initial codes. We then searched through the codes for emergent themes describing the challenges and recommendations. The themes were then reviewed, renamed, and summarized as presented in this manuscript.^16^

## Results

We identified 163 articles discussing challenges with integrating digital technologies in public health and/or making recommendations on ways to surmount these challenges. Table 2 presents an overview of the characteristics of the 163 included articles. The majority of the publications (53.4% (87)) were review-type articles, 63.2% (103), were written by first authors based in North America (United States of America and Canada), and 64.4% (105) were published between 2015 and 2020.

**Table 2.** Characteristics of included publications.

Characteristics	Frequency (n = 163)	Percent (100%)
**Article type**		
Commentary	29	17.8
Editorial	17	10.4
Report	16	9.8
Review	87	53.4
Others^a^	14	8.6
**Country of the first author**		
USA	84	51.5
UK	21	12.9
Intergovernmental Organization	7	4.3
Canada	18	11.0
Australia	8	4.9
Switzerland	5	3.1
Others^b^	20	12.3
**Continent**		
Asia	6	3.7
Europe	41	25.2
Transcontinental Organization	5	3.7
North America	103	63.2
Oceania	8	4.9
**Years of publication**		
2000–2004	9	5.5
2005–2009	12	7.4
2010–2014	37	22.7
2015–2020	105	64.4

^a^ Includes workshop and conference summary recommendations, policy statements, and glossary documents.

^b^ It includes Israel, Ireland, Norway, Italy, Cyprus, and Germany among others.

Emergent from our thematic analysis were two broad categories of challenges faced in digital public health: technical and nontechnical challenges. Technical challenges were defined as those related to the technologies and technologically related development and implementation processes relevant to integrating digital tools in public health. Nontechnical challenges referred to other issues pertinent to public health generally but that were emphasized when integrating digital technologies. While these demarcations are somewhat arbitrary and overlapping, we found it an intuitive framing to identify dominant perspectives in the literature. Technical challenges were discussed mainly from an engineering and technical perspective, while nontechnical challenges were discussed from a public health and social science perspective. Overall, nontechnical challenges were more prominently highlighted as key barriers to the development and implementation of digital public health interventions. Both technical and nontechnical challenges were often described as being interrelated and mutually reinforcing.

### Nontechnical challenges

#### Ethical and legal issues

Articles highlighted ethical challenges to the use of digital technologies in public health related to data privacy, confidentiality, ownership, and security.^17–21^ Protecting individuals’ right to personal privacy and the confidentiality of their health information, preventing re-identification of individual-level data, and clarifying data ownership were described as challenges for the data integration required for public health functions.^22–27^ Related to data security, articles highlighted inconsistent data encryption standards especially across jurisdictions^27–30^ and varied ethical and legal traditions as a key barrier.^30^ The risk of unauthorized data access and concerns about (mis)appropriation of data beyond consented uses, including for research and commercial purposes, were also emphasized.^26,31,32,35,33 34^ Additional ethical barriers include the potential identification of individual disease risks and exposures through digital technologies without clear remedies,^36–38^ perpetuation of stigma through data collection methods that do not acknowledge the existing context.^24,39,40^ Further concerns were expressed on the inherent shift in the responsibility of disease prevention and care from clinical and public health institutions to individuals as digital interventions may fail to acknowledge core structural issues that manifest as public health issues.^41–43^

#### Health equity

Closely related to the ethical issues, articles noted that numerous digital interventions in public health have not adequately considered health equity in their design.^35,44–49^ The most frequently reported health equity issue concerned the digital divide as expressed in two main ways. First, articles described disparities in access to digital technologies (including Internet services, smartphones, and computer systems) and use of digital health services based on gender, sexual orientation, age, education, ethnicity, urbanicity, and income.^17,35,41,46–48,50–62^ Further, disparities in digital health literacy, including users’ ability to understand and act on available digital information were highlighted as another major equity challenge.^35,38,48,49,53,55,56,58,60–70^

Further, digital technologies in public health may disproportionately benefit already privileged groups in society, and exclude marginalized populations.^26,33,46,50,71,72^ This may be further exacerbated through the economic incentive to target high-income users with commercial digital health interventions.^71^ Other equity challenges described include disparities in the impact of overlooked potential consequences of digital technologies, such as data privacy breaches, data misuse, and biased algorithms leading to the perpetuation of stigma on marginalized populations.^21,26,33,35,38,40,73,74^ Widespread lack of digital health equity implementation frameworks and rigorous evaluation of the equity impacts of these digital technologies may further complicate equity challenges.^36,44,45,73^

#### Policy and governance challenges

Articles described challenges with policy keeping pace with evolving digital technologies in public health,^20,23,26,40,50,63,64,75–79^ resulting in reactive policies that are restrictive (often limiting data exchange), inadequate to support the development and implementation of digital technologies in public health,^20,24,50,77,80^ or not aligned with prevailing needs.^54^ Governance challenges in supporting interjurisdictional digital interventions were also highlighted,^20,22,29,30,50^ especially for technologies not requiring in-person attendance. The transdisciplinary nature of digital public health interventions and the diversity of regulatory bodies involved in their development and implementation further reinforces the inadequacy of existing policy interventions.^22,32,54,77^

#### Paucity of high-quality evidence

The paucity of scientifically rigorous evidence on the real-world effectiveness of digital public health interventions in different contexts and for different populations was described as a significant challenge.^33,35,36,40,45,46,77,81–87^ Articles noted the inadequacy of cost–analysis evidence, specifically the costs of these interventions to individuals and health systems,^36,64,77^ and the mostly retrospective use of data in existing research that may be biased or have missing data.^87^ Further, the iterative and fast-paced evolution of digital technologies is not matched by current research methods which are often rigid and slow to adapt.^27,36,43,45,77,82,83,88,89^ There is also a lack of widely agreed-upon standards for assessing outcomes^55,90^ and reliable outcome measures that extend beyond user engagement metrics.^35,48,77,82,83,86,90,91^ Further, interventions were frequently implemented on a small scale and their associated evaluations were described as poorly generalizable.^48,92^ Reliance on non-validated user-provided information was also said to reduce chances of successful follow-up in cases of attrition^93^ and reduce the representativeness of data.^83,93^ Lastly, many digital public interventions were said not to be guided by known theory and lack sufficient documentation of their processes, making them difficult to replicate.^43,94^

#### Resource and economic interest barriers

The lack of long-term economic investments required to maintain essential infrastructure and human resources were said to complicate the sustainability of digital innovations in public health,^21,50,54,63,64,84,94,95^ especially with limited public health funding.^19,26,49,50,78,96–102^ Difficulties establishing clear cost–benefit outcomes and accountability strategies were also identified.^48,97,101,103^ These difficulties were reported to be worse among public health practitioners working in resource-limited settings.^73^ Current innovations in public health were described as driven by commercial interests of for-profit organizations, often targeting populations serving these interests, worsening existing health disparities, and limiting opportunities for widespread impact in line with public health principles.^2,50,71,76^

#### Disinformation and misinformation

Articles described digital media, including social media as susceptible to the amplification of false and poor-quality health information, impacting health promotion efforts online.^33–35,56,69,104,105^ The nature of such platforms that leverage user-generated content^35,51,62,106^ with relative anonymity^51,107^ and unclear guidelines^43,105^ were said to spread unverified and often questionable information.^34,43,51,52,108^ Further, information overload on the public and the burden of health-information verification responsibilities placed on individuals was said to potentially worsen existing health disparities^35,80,108,109^ given that the capacity to verify the information is dependent on digital health literacy.^69,110^ Overall, the authors suggest that these challenges result in poor health behaviors and over-medicalization of common health problems,^56,62,77,80,108^ with public health practitioners struggling to control the messaging within these fast-moving media platforms.^95^

#### Technological optimism

The overly enthusiastic assumption that digital technologies provide a catchall solution to public health challenges without due consideration of alternative approaches and foundational principles of public health was noted.^18,38,40,111^ Articles suggested that digital technologies may propagate a reductionist view on public health that ignores underlying social, economic, environmental, and commercial determinants of health, reducing their effectiveness.^17,38,41,42,87,106,112^ Further, inherent biases in assumptions and algorithms facilitating technologies like artificial intelligence, big data, and social media in public health were reported to potentially make them counterproductive to public health goals.^21,28,36,47,113,114^ These technologies were said to sometimes draw false associations and conclusions, perpetuate stigma, and widen health disparities.^28,36,113,114^

### Technical challenges

#### Unreliability of available data

The heterogeneous nature of data sources and large volumes of data including epidemiologic, surveillance, and health services data required for public health services gathered across various health systems^89,113,115–117^ without broad standards for reporting and consistency was emphasized as a problem of data reliability.^23,37,85,89,116,118–122^ Further, data quality issues, including incomplete data, were said to be a challenge,^24,40,120,123,124^ especially for big data in public health surveillance and research, where noisy data (i.e. poor quality data) reduces efficiency.^89,116,125^ Further, the unreliability of unvalidated or anonymous user-generated and/or observational data^52,56,79,80,83,91,92,118,126–128^ were said to bias reports. For example, reports on social media could misrepresent the general public health situation, as significant portions of the population, including people suffering disparities in access to digital technologies, are excluded from the data.^21,38,40,46,73,85,89,91,101,116,125,129,130^

#### Fragmented, isolated, and unsustainable systems

A major challenge highlighted in the literature was the lack of coherent, coordinated, and integrated digital health architecture and coding standards across public health and allied agencies at the institutional and country-level, with proprietary systems that must be interoperable to support public health goals.^22,23,26,28,50,103,121,131,132^ This partly results from privacy and confidentiality concerns,^22,37^ and infrastructure/capacity gaps.^22,123^ Further disease and/or area-specific funding for digital interventions^99,131^ and *ad hoc* design of these systems were said to result in multiple isolated systems developed by different agencies and technology providers.^4,27,31,133^ The development and implementation of piecemeal interventions with limited funding that are sustainable and unable to harness economies of scale required to drive down operational costs were said to be characteristic of this problem.^26^ Insufficient coordination and administrative barriers between agencies at all levels resulted in duplicated investments and low motivation for long-term investments.^27,97,134^

#### Leadership and health workforce capacity-building gaps

We found that strong technical leadership is crucial to facilitating the transformational change required to navigate complex, multifaceted, multi-stakeholder contexts critical for integrating digital technologies in public health.^17,96,97^ Public health practitioners have been noted to lack the technical training and experience necessary to make strategic decisions about information technologies and implement these systems.^22^ The public health workforce has also been noted to lack the requisite technical expertise to benefit fromthe potential advantages of digital technologies in public health.^22,37,43,63,64,73,98,99,123,131,135^ Public health agencies are said to have challenges with engaging and retaining highly demanded tech-savvy public health analysts.^37,40,99,123^ New skills will be required in the public health workforce, including data manipulation, data mining, business analysis, project management, and social media communication, which is outside the current job descriptions of most public health workers.^25,37,54,73,110,136^ The public health workforce has not been supported to develop these capacities at the same pace as the push for development of the technologies required in digital public health. Health workers have also shown limited trust in digital technologies and a reluctance to learn new digital skills given concerns about the potential adverse effects on the public's interaction with health systems and the feeling of being unprepared to engage new systems.^22,133,137^

#### Infrastructure gaps

Public health increasingly relies on complex, high-volume data from heterogenous sources. Ensuring the promise of better public health precision in a digital era imposes new infrastructure requirements to enable operations.^22,73,118,132,138,139^ More robust computing infrastructure,^25,64,132,138^ including high-bandwidth, low-latency computer networks and clusters of machines for computation^19,25,60^ are required to take advantage of high-volume data. This was mostly emphasized in the use of big data and artificial intelligence for public health surveillance, epidemiology, and research. Capacity for ongoing maintenance of infrastructure and access to repair parts also constitute a barrier.^98^ Many public health agencies lack access to such computing or IT power. Further, in resource-limited settings, unreliable power supply is a complicating factor.^36,98^ In addition to providers, public health service users also require access to computers, smartphones, and Internet services, which are often disparately distributed along socioeconomic gradients. These infrastructure requirements are closely related to funding and human resource needs, as the development of infrastructure needed to implement effective information systems has been slow, especially in financially limited local health departments.^22^

#### Lack of clear consensus on operations standards

The lack of clear consensus on operations standards demonstrates the nascent nature of digital technologies in public health.^135^ Considering the heterogeneity of data required for public health functions, reporting standards are required to effectively pool data; however, these standards are yet to be developed.^22,36,134,140^ Standards in vocabulary and health information exchange and coordination are lacking, resulting in suboptimal interoperability of varied systems relevant to public health functions.^4,22,64,103,141^ Standards for evaluating the impact of digital technologies on health outcomes are also suboptimal.^55^ Where there are standards, public health professionals have been shown to have suboptimal awareness of existing data reporting standards^22^ and data quality requirements across systems.^36,64,108,136^

#### Suboptimal design and implementation of digital technologies

Many digital technologies in public health are characterized by overenthusiastic and sometimes complex designs that neglect basic foundations of digital health, including attention to users’ needs rather than needs perceived by the implementers, and implementation of interventions that are based on proven behavioral theories.^45,49,51,87,142–144^ Further, the focus on using digital interventions for data collection, without due consideration to the value they might offer to frontline health providers and other user potentially limits engagement.^30,142,145^ User contexts have also been oversimplified and not well accounted for in the development of digital tools, with limited evaluation of the usability and acceptability of the tools among targeted users.^122,142^

### Mapped solutions to overcome technical and nontechnical challenges

Potential solutions, as described in the literature, are summarized in Table 3. We identified seven overarching themes suggested to surmount technical and nontechnical challenges in digital public health.

**Table 3.** Challenges to integrating digital technologies in public health and mapped solutions as identified in the literature.

Challenges	Mapped recommendations
**Nontechnical challenges**	
Ethical, legal, and privacy issues	Establishment of ethical, legal, and regulatory frameworks^a^ Transparent communication strategy to promote public trust^a^ Open-source IT architecture with appropriate safeguards Research on additional privacy methods^a^ Partnerships and collaboration including members from affected communities^a^ Unique digital identifiers Widespread stakeholder engagement (going beyond consultation to ensure close collaboration)^a^
Paucity of high-quality evidence	Articulation of a clear research agenda by government agencies, public health researchers, and practitioners^a^ Creation of new and adaptive research and evaluation methods^a^ Digital interventions implemented based on proven behavior theories Integration of evaluation activities at all stages of implementation^a^ Partnerships between researchers and digital public health practitioners^a^
Health equity	Health equity-focused design of digital public health interventions Deliberate action to foster digital literacy Equity-focused evaluation of digital health interventions^a^ Establishing communities of practice addressing health disparities^a^ Integrate digital interventions in universal health coverage strategies Health equity investments^a^ Publicly available digital access points
Policy and governance challenges	Transdisciplinary partnerships^a^ Coordinate frameworks to ensure adherence to standards and interoperability^a^ Data governance and accountability frameworks^a^ Advisory bodies at national, sub-national, and local levels Transparent public trust frameworks^a^
Resource barriers	Inter-sectorial approach that engages a wide range of stakeholders^a^ Evidence of returns on investments in digital health interventions^a^ Establishment of collective funding streams for digital health through public health agencies^a^ Focus on long-term funding needs across project life cycles Shared financial risks through public–private partnerships^a^
Disinformation and misinformation	Proactive communication plans and policies to counter destructive posts^a^
Technological optimism	Needs-based services Robust research to support digital interventions^a^ Balanced investment in innovation and ongoing investment in established methods^a^
**Technical challenges**	
Unreliability of available data	Standardized reporting systems Inter-sectorial partnerships^a^ Artificial intelligence assisted reporting Research on data quality assurance methods^a^
Fragmented, isolated, and unsustainable systems	Shared digital architecture and standards^a^ Clear reporting standards and dissemination of such standards^a^ Create interoperability requirements for digital health interventions Early sustainability and integration planning Establish health information exchange systems
Leadership and health workforce capacity-building gaps	Political commitment to integrate digital technologies Health workforce training^a^ Health workforce redesign including updated job descriptions^a^ Interdisciplinary communities of practice and knowledge exchange
Infrastructure gaps	Long-term resource planning and economic investments^a^ Collaborative infrastructure building and resource pooling^a^ Capacity building to complement infrastructure investments^a^
Lack of clear standards	Create normative standards/regulatory frameworks^a^ Compile data dictionaries and repositories of digital public health tools
Suboptimal design and implementation of digital technologies	Needs-based digital health services Implementation based on proven behavioral theories

^a^ Indicates recurring recommendations to address multiple challenges identified.

#### Political commitment

The importance of political commitments of governments and leaders to implement digital public health strategies across jurisdictions was emphasized.^22,121,146^ Political commitment is important to surmount resource barriers and foster the development and implementation of ethical, legal, and governance frameworks required for the seamless development of digital public health systems.^66,97,147,148^ The argument for political commitment is premised on potentially improved public health outcomes and reduced health costs through the provision of accurate and timely aggregate-level data to support decision making.^22^ This multi-institutional commitment must include a wide range of stakeholders including the government, private sector organizations, nonprofit organizations, engineers, innovators, academia, research institutes, health care providers, and other government institutions.^97,121^

#### Intersectoral transdisciplinary partnerships

Intersectoral and transdisciplinary partnerships between governments, academics, public health practice, and industry partners were recommended to maximize currently available resources through resource pooling, shared expertise, and shared priority setting across jurisdictions.^27,53,145,148^ Such partnerships were also deemed important for creating coherent and coordinated interoperability, governance, and research frameworks, and garnering required resources, knowledge, and capacities to harness the potential of digital public health interventions.^22,97,98,117,149^ Such partnerships can help agencies and organizations leverage their comparative advantage and circumvent their own restraints.^21^ The importance of these partnerships in securing political commitment to ensure that digital interventions move beyond pilot interventions to bringing them at scale was also emphasized.^97,148^

#### Economic investments

Significant economic investments were shown to be relevant to the development and implementation of digital public health. Investments must be planned for sustainability, ensuring funding across the project life-cycle and beyond pilot phases.^40,84,148–150^ Phased planning of interventions with funding for each cycle of implementation was also suggested.^22,145^ Opportunities to share risks through integrated planning and pooled resources across the public and private sectors must be explored.^22,150^ The importance of investing in interventions that improve the health equity outcomes of populations, including securing access to Internet services and publicly available digital access points, were also discussed.^21,151^ Such investments must be backed by evidence of the cost-effectiveness of interventions which also require investments in research and evaluation.^22,40,77,134,146^

#### Standardized ethical, legal, and regulatory frameworks

Establishing standardized ethical, legal, and regulatory frameworks was recommended as a pivotal aspect of facilitating the development and implementation of digital public health interventions. These frameworks should ensure a balance between innovation and ethics and social justice principles of public health.^66,77,148^ Standardized reporting systems and requirements,^22,77,145^ data quality standards, and shared digital architecture^22,23,77,144,145^ were suggested as mechanisms to ensure reliable and interoperable data systems to foster improved use of data for decision making. Articles highlighted the importance of widespread partnerships, coordinating frameworks, advisory, and oversight bodies at all levels.^22,45,145,149,152^ Members of the public served by these interventions should be involved beyond just consultation, ensuring their collaboration in the development and implementation of interventions.^67^ This also involves wide-ranging collaboration and action among government agencies, in partnership with other stakeholders, to ensure that data governance and accountability frameworks are adaptable, proactive, and person-centered.^23,40,66,145,146,153,154^

#### Health workforce capacity building

Building capacity of the health workforce was suggested to help surmount both technical and nontechnical challenges. Capacity building is needed for workers to proactively identify digital infrastructure gaps, take advantage of available infrastructure, and maximize the impact of digital public health interventions.^19,98,146^ Ensuring the integration of digital technologies into existing public health training curricula can reduce data quality issues, inform design of relevant frameworks, and improve the capacity to manage ever-increasing volumes of health data.^19^ Articles suggest that such curricula may be made open through communities of practice and interdisciplinary workforce exchanges to foster equitable knowledge uptake.^21,98^ Further, a deliberate effort to integrate diversity into health workforce capacity-building plans is also recommended to foster equity in the design and implementation of digital public health interventions.^73,98^ Others recommend updated job descriptions during recruitment that seek to bridge diverse fields involved in digital public health, including data science, engineering, health monitoring, and public health.^4,116^

#### Adaptive and comprehensive research and evaluation

The development and implementation of digital public health interventions was reported to require the articulation of a clear research agenda that focuses on generating high-quality evidence to support ongoing investment in the field at all local, national, and international levels.^22,38,40,48,134,145,146^ New methodology suitable for the peculiar pace of development and application of digital technologies, with appropriate measures of health outcomes, are also required to advance research and evaluation in digital public health.^21,27,40,48,58,77,146,149,152^ Mechanisms for actively sharing these methods were emphasized, including networks and inter-sectorial communities of practice.^27^ Research and development of new privacy, data sharing, and governance frameworks relevant to digital public health were also recommended.^30,40,117,136,149^ Further, recommendations involved integrating research and evaluation in all stages of development and implementation of digital interventions.^77^ These recommendations also highlight the importance of reporting outcomes of digital interventions disaggregated by factors including gender, race, ethnicity, age, and socioeconomic status to inform adaptations to improve health equity.^44,72,134,145^

#### Transparent communication and public engagement

Having a clear and transparent communication strategy was recommended in the literature to build trust and engagement between implementers and public health users. Transparency is recommended between stakeholders to ensure clear ownership and governance structures for fair data access, maintenance of security, and inclusion.^26,66,98,124,138,149^ Open science, equity-based frameworks were also recommended with a focus on including outcomes for marginalized populations in publications.^23,111^ Proactive public communication strategies are also recommended to mitigate the risk of public mistrust of digital interventions within this trust model. Such strategies must communicate clear and evaluable benefits of digital public health interventions, including data sharing, and proactively address the potential for misinformation to the public (including health care workers).^21,26,30,66,69,77,86,152^ Engagement strategies must also consider populations that suffer disparities to foster trust and ensure health equity.^17,98^

## Discussion

This scoping review provides a high-level description of the challenges faced by digital public health practitioners and identifies broad-level recommendations to address these challenges. Our review expands on an existing review^2^ and describes multiple, complex, and interrelated technical and nontechnical challenges in digital public health. Nontechnical challenges regarding ethics, paucity of evidence, equity, policy and governance concerns, and resource barriers dominate the discourse. Technical challenges including unreliability of data, fragmented, isolated, and unsustainable systems, leadership and health workforce capacity gaps, infrastructure gaps, and a lack of clear standards also constitute significant barriers to integration of digital technologies in public health. Seven recurring recommendations were identified to address these challenges, including securing political commitment, intersectoral transdisciplinary collaboration, economic investments, standardized ethical, legal, and regulatory frameworks, adaptive and comprehensive research and evaluation, health workforce capacity building, and transparent communication and public engagement.

**Figure 2.**
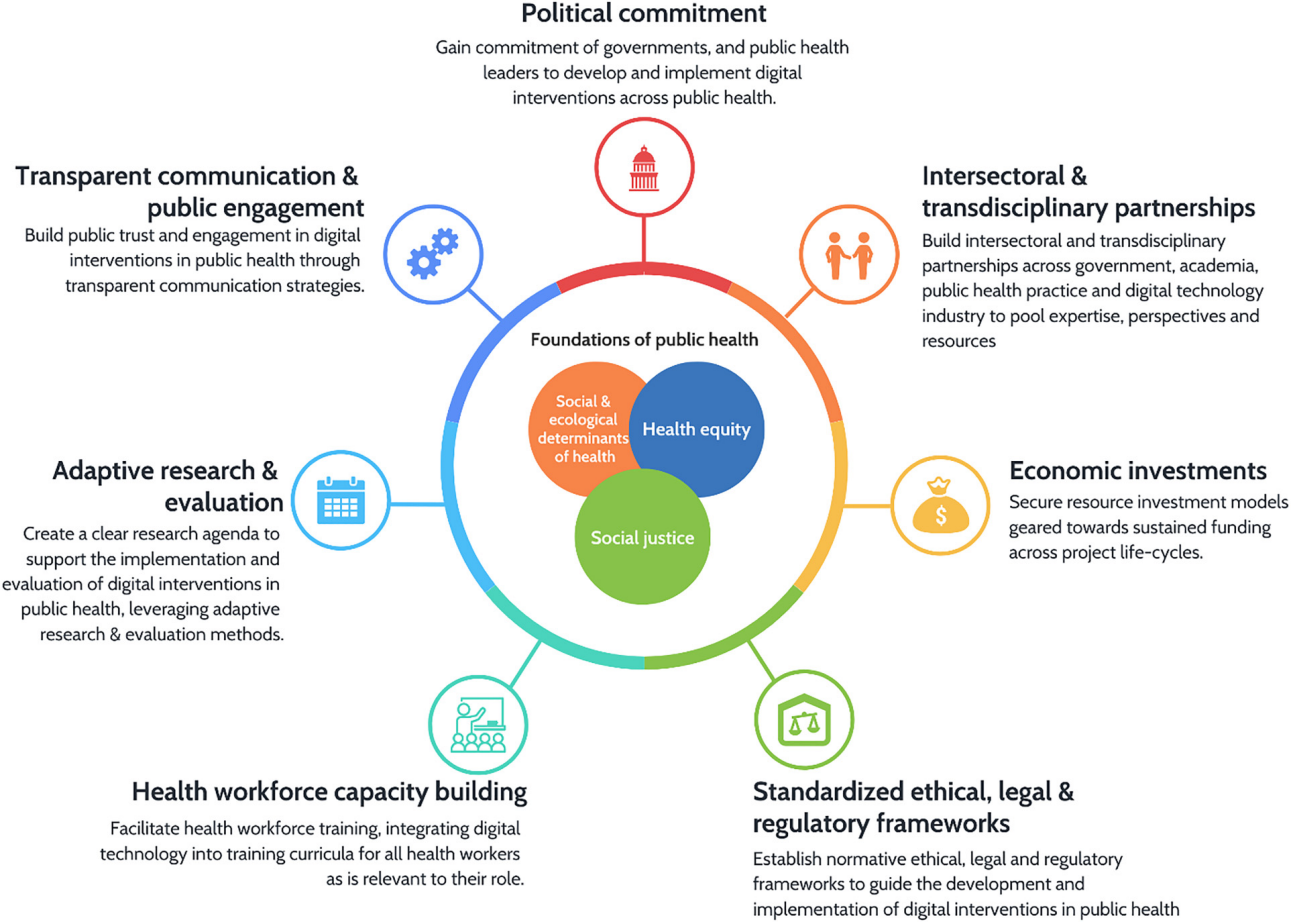
Mutually reinforcing pillars of digital public health.

These challenges have been well documented in reference to specific domains of public health.^6,7,115,155^ For example, privacy and security issues have been documented in relation to public health surveillance. Findings from our review underscore the complexity involved with digital public health and the interrelated challenges previously described in other studies.^2^ The findings of our review also underscore cross-cutting themes related to the ethical integration of digital technologies across the domains of public health as described in most of the challenges identified. Further, our high-level description of the challenges extends the literature by mapping out recommended solutions to these challenges, summarily providing actionable strategies that public health policy makers, practitioners, and researchers may explore to foster cohesive and effective development of digital public health. These identified solutions which we refer to as key pillars of digital public health, validate suggestions made by the European Public Health Association (EUPHA).^2^ These pillars must be closely considered in tandem with the foundational principles guiding public health practice to create a clear agenda for the ongoing development of the field (Figure 2).^14^

The COVID-19 pandemic has fostered an unprecedented level of political commitment to identify strategies to optimally harness the potential of digital technologies in public health.^156^ This is a crucial opportunity for policy makers, public health practitioners, and researchers to envision digital public health in a cohesive manner.^157,158^ Leveraging these key pillars as a central aspect of a digital public health strategy can be an effective starting point to ensure the successful development of the field. In envisioning and implementing digital technologies, the importance of aspiring for deep-rooted inter-sectorial transdisciplinary partnerships across traditional public health practices, and nontraditional fields including engineering, computer science, ethics, and behavioral sciences cannot be overstated.^4,6,136^ The defining feature of these transdisciplinary partnerships is the transcendence of disciplinary perspectives and boundaries (across academia and industry) in a systemic manner that involves practical participatory engagement with real-world problems to create feasible, socially acceptable, effective, and sustainable solutions.^159,160^ However, we must note that achieving transdisciplinary partnerships will be challenging given differences in vocabulary across disciplines and varied epistemological viewpoints that guide practice and research.^159^

Moreover, in creating these partnerships, we must clarify the role of private sector players in digital public health.^7^ Existing concerns about commercial interests have been a source of public distrust of digital public health interventions and must be addressed using established principles guiding public health practice.^21,73,121,147^ Further, standardized ethical, legal, and regulatory frameworks are required to ensure seamless, secure data exchanges between health agencies and authorities. Creating these normative frameworks will be complex, as the literature has highlighted multiple barriers to their establishment. For example, the interjurisdictional coverage of digital public health approaches implies that there must be political engagement to facilitate these frameworks.^146,161^ Yet, resistance to these new approaches within public health and the tendency to suppress data especially with suboptimal interventions, introduce additional challenges that must be navigated.^3^ The speed of technological development must also be considered in these frameworks.

Standardized regulatory frameworks and policies centered on foundational public health principles must be informed by evidence. There is a need for adaptive research and evaluation frameworks, as well as a comprehensive agenda to guide the development and implementation of digital interventions in public health. Such adaptive research must respond to the implementing context and balance research and rigor with a need for data especially on technology that is rapidly evolving.^21^ An iterative approach that leverages a wide range of methodologies has been recommended to ensure that these divergent concerns are addressed.^77^ Further, adaptive health technology assessment models have been suggested in the context of public health emergencies like COVID-19.^162^ However, given the evolving nature of digital technologies in public health, it is recommended that adaptive research and evaluation methods should the standard.^162^ Further, for pragmatic policies to be made and implemented appropriately, public health policy makers, practitioners, and researchers will require continued education on new working methods in a digital environment. Considering low public trust in digital technologies generally, there is a need for proactive communication and engagement strategies to foster the growth of digital public health. Such proactive strategies are also necessary to stem the tide of misinformation and disinformation facilitated through digital technology.^52,154^ There is an ethical imperative to implement such strategies as misinformation and disinformation amplified through digital technology has significant public health consequences.

### Limitations

Given the broad scope of this review, a more in-depth discussion of the challenges and recommendations was not practical. We focus here on broad-level challenges which may inadvertently leave out some of the more specific complexities within domains of public health and digital technologies employed. Further, considering that this is a sub-analysis of a larger scoping review to define digital public health, we acknowledge that the search strategy may not be specific enough and may have inadvertently excluded some relevant literature that provides provide an in-depth understanding of the challenges of integrating digital technologies in specific domains of public health. However, given the high-level aims of the review, we remain confident that the breadth of literature included gives a clear understanding of the broad challenges inherent in the field. Finally, our search was conducted in 2020. As such, additional insight may be available in more recent literature exploring digital technologies in the public health response to COVID-19.^163,164^ However, we remain confident that the broad challenges and strategies identified in this review remain relevant in our current context.

## Conclusion

Despite growing interest in digital public health, a plethora of complex, evolving, and interrelated technical and nontechnical challenges hinder the ongoing development and implementation of digital interventions in public health. To foster a stronger approach to the development and implementation of digital public health, consolidated efforts are required to facilitate the comprehensive integration of digital technologies in public health at a scale that improves health outcomes for all. These efforts may leverage the seven key pillars identified in this review, including securing political commitment, inter-sectorial collaboration, economic investments, standardized ethical, legal, and regulatory frameworks, adaptive and comprehensive research and evaluation, health workforce capacity building, and transparent communication and public engagement. These key pillars may be considered as a roadmap to inform stronger and more evidence-based development of digital public health interventions.

## Notes

1. In some of the papers, the CPHA dimensions may not be explicitly stated. Using our own judgement and knowledge of public health will be useful in determining the relevance of the paper to this study. For example, a digital health intervention may be described in “disaster preparedness” which is not directly described in the framework, but it aligns with emergency response. This kind of paper can be included if the other inclusion criteria are met.

## Appendix

### Appendix 1. Search strategy

Database; Ovid MEDLINE(R) and Epub Ahead of Print, In-Process, and Other Non-Indexed Citations, published from 2000 to 1 June 2020

Search was conducted on 1 June; all languages included.[Table table4-20552076221102255]

**Table table4-20552076221102255:**

Searches
1. (mhealth or m-health).mp. [mp = title, abstract, original title, name of substance word, subject heading word, floating sub-heading word, keyword heading word, organism supplementary concept word, protocol supplementary concept word, rare disease supplementary concept word, unique identifier, synonyms]
2. virtual health.mp.
3. mobile health.mp.
4. (ehealth or e-health).mp. [mp = title, abstract, original title, name of substance word, subject heading word, floating sub-heading word, keyword heading word, organism supplementary concept word, protocol supplementary concept word, rare disease supplementary concept word, unique identifier, synonyms]
5. online health.mp.
6. internet-based health.mp.
7. computer-based health.mp.
8. digit*.tw.
9. electronic health.tw.
10. health informatics.mp.
11. web-based*.tw.
12. telehealth.mp.
13. Telemedicine/
14. Social Media/
15. predictive algorithms.mp.
16. machine learning methods.mp.
17. Machine Learning/
18. Big Data/
19. public health*.tw.
20. Health Promotion/
21. health prevention.mp.
22. health protection.mp.
23. Health Policy/
24. health determinants.mp.
25. Population Surveillance/
26. health evaluation.mp.
27. health economics.mp.
28. Risk Assessment/
29. Epidemiology/
30. community health.mp.
31. emergency preparedness.mp.
32. emergency response.mp.
33. Health Equity/
34. Social Justice/
35. social determinants.mp.
36. Artificial Intelligence/
37. digital public health.mp.
38. digital health.mp.
39. digitalization.mp.
40. digital tools.mp.
41. digital technologies.mp.
42. web-based health.mp.
43. 1 or 2 or 3 or 4 or 5 or 6 or 7 or 9 or 10 or 12 or 14 or 15 or 16 or 18 or 36 or 37 or 38 or 39 or 40 or 41 or 42
44. 19 or 20 or 21 or 22 or 23 or 24 or 25 or 26 or 27 or 28 or 29 or 30 or 31 or 32 or 33 or 34 or 35
45. 43 and 44
46. ((mhealth or m-health or virtual health or mobile health or (ehealth or e-health) or online health or internet-based health or computer-based health or electronic health or health informatics or telehealth or Social Media or predictive algorithms or machine learning methods or Big Data or Artificial Intelligence or digital public health or digital health or digitalization or digital tools or digital technologies or web-based health) and (public health* or Health Promotion or health prevention or health protection or Health Policy or health determinants or Population Surveillance or health evaluation or health economics or Risk Assessment or Epidemiology or community health or emergency preparedness or emergency response or Health Equity or Social Justice or social determinants)).tw.
47. limit 46 to (english language and yr = "2000 -Current”)
48. Public Health/
49. public health ethics.mp.
50. 20 or 21 or 22 or 23 or 24 or 25 or 26 or 27 or 28 or 29 or 30 or 31 or 32 or 33 or 34 or 35 or 48 or 49
51. 43 and 50
52. ((mhealth or m-health or virtual health or mobile health or (ehealth or e-health) or online health or internet-based health or computer-based health or electronic health or health informatics or telehealth or Social Media or predictive algorithms or machine learning methods or Big Data or Artificial Intelligence or digital public health or digital health or digitalization or digital tools or digital technologies or web-based health) and (Health Promotion or health prevention or health protection or Health Policy or health determinants or Population Surveillance or health evaluation or health economics or Risk Assessment or Epidemiology or community health or emergency preparedness or emergency response or Health Equity or Social Justice or social determinants or Public Health or public health ethics)).tw.
53. limit 52 to (english language and yr = "2000 -Current”)
54. 1 or 2 or 3 or 4 or 5 or 6 or 7 or 10 or 12 or 14 or 15 or 16 or 18 or 36 or 37 or 38 or 39 or 40 or 41 or 42
55. 50 and 54
56. ((Health Promotion or health prevention or health protection or Health Policy or health determinants or Population Surveillance or health evaluation or health economics or Risk Assessment or Epidemiology or community health or emergency preparedness or emergency response or Health Equity or Social Justice or social determinants or Public Health or public health ethics) and (mhealth or m-health or virtual health or mobile health or (ehealth or e-health) or online health or internet-based health or computer-based health or health informatics or telehealth or Social Media or predictive algorithms or machine learning methods or Big Data or Artificial Intelligence or digital public health or digital health or digitalization or digital tools or digital technologies or web-based health)).tw.
57. limit 56 to (english language and yr = "2000 -Current”)
58. Public Health Surveillance/
59. 20 or 21 or 22 or 23 or 24 or 26 or 27 or 28 or 29 or 30 or 31 or 32 or 33 or 34 or 35 or 48 or 49 or 58
60. 54 and 59
61. ((mhealth or m-health or virtual health or mobile health or (ehealth or e-health) or online health or internet-based health or computer-based health or health informatics or telehealth or Social Media or predictive algorithms or machine learning methods or Big Data or Artificial Intelligence or digital public health or digital health or digitalization or digital tools or digital technologies or web-based health) and (Health Promotion or health prevention or health protection or Health Policy or health determinants or health evaluation or health economics or Risk Assessment or Epidemiology or community health or emergency preparedness or emergency response or Health Equity or Social Justice or social determinants or Public Health or public health ethics or Public Health Surveillance)).tw.
62. limit 61 to (english language and yr = "2000 -Current”)
63. 61 not “trial”.mp. [mp = title, abstract, original title, name of substance word, subject heading word, floating sub-heading word, keyword heading word, organism supplementary concept word, protocol supplementary concept word, rare disease supplementary concept word, unique identifier, synonyms]
64. 63 not “cross-sectional”.mp. [mp = title, abstract, original title, name of substance word, subject heading word, floating sub-heading word, keyword heading word, organism supplementary concept word, protocol supplementary concept word, rare disease supplementary concept word, unique identifier, synonyms]
65. limit 64 to (english language and yr = "2000 -Current”)

### Appendix 2. Framework for full-text review

1. Was access gained to the full paper of the citation
  □ Yes, full paper accessed^1^
  □ No, full text could not be downloaded^2^
  □ Only abstract available or full article less than 500 words^3^

If yes to step 1, move to step 2, else stop the review and indicate reason for exclusion

2. Is the full text in English?
  □ Yes^3^
  □ No^4^

If option 3 (yes) is selected, move to step to the next step, else stop the review and indicate reason for exclusion as “Language not English”

3. Does the paper broadly conceptualize and/or analyze a digital health intervention/application from any public health perspective (as described in the CPHA framework)? ^1^
  □ Yes, it is a commentary/narrative review/systematic review broadly applying digital health in public health^5^
  □ Yes, it presents a conceptual analysis of digital health in public health, but as a section of a broader public health or digital health discussion^6^
  □ No, it is a primary study or presents a report of a specific digital health intervention^7^
  □ No, it is a commentary focusing entirely on describing a specific digital health intervention^8^
  □ No, it is a systematic review that presents a glossary of specific digital health interventions^9^
  □ No, it discusses digital health **entirely** from a clinical perspective^10^
  □ Can't tell^11^

If option 5 or 6 is selected, including the paper, else exclude and indicate “not conceptual” for options 7–9, or “clinical/technical perspective” for option 10. If option 11 is selected, indicate “maybe” and discuss with the co-reviewer.
